# Intramucosal Carcinoma of the Appendix Arising from Traditional Serrated Adenoma

**DOI:** 10.1155/2015/297450

**Published:** 2015-04-22

**Authors:** Carlos Augusto Real Martinez, Júlia Cutovoi, Debora Helena Rossi, Luciana Rodrigues Meirelles, Maria de Lourdes Setsuko Ayrizono, Raquel Franco Leal, Cláudio Saddy Rodrigues Coy

**Affiliations:** ^1^Faculdade de Ciências Médicas, Universidade Estadual de Campinas, Rua Tessália Vieira de Camargo, No. 126, Cidade Universitária “Zeferino Vaz”, 13083-887 Campinas, SP, Brazil; ^2^Gastrocentro, Universidade Estadual de Campinas, Rua Carlos Chagas, No. 420, Cidade Universitária “Zeferino Vaz”, 13083-878 Campinas, SP, Brazil

## Abstract

*Introduction*. Serrated adenomas of the appendix are rare and usually found during appendectomy or autopsies. The preoperative diagnosis of these tumors is uncommon. This report describes a case of a sessile serrated adenoma located in the appendix diagnosed by a screening colonoscopy and successfully treated by laparoscopic removal. *Presentation of Case*. An 86-year-old woman underwent colonoscopy to investigate the cause of her diarrhea, weight loss, and anemia. During the colonoscopy, an expansive and vegetating mass of 1.5 cm in diameter was identified, protruding through the appendicular ostium with slightly lateral growth to the cecum. The patient was referred for laparoscopic surgical resection due to the location of the lesion, which did not allow its removal by colonoscopy. She underwent wedge removal of the cecum without complications and was discharged on the 4th postoperative day. Histopathological examination showed the presence of a sessile serrated adenoma with an intramucosal adenocarcinoma. The patient is currently well one year after surgery, without endoscopic signs of relapse. *Conclusion*. Despite serrated adenomas being a possibility rarely described in appendix it should be recognized and properly treated because it is presenting a higher risk of cancer.

## 1. Introduction

Sessile serrated adenoma (SSA) is a relatively recently described entity. SSA is a specific type of adenoma more commonly located on the right side of the colon and rarely in the appendix [1–3]. Studies have demonstrated that SSA develops from the serrated pathway of colorectal carcinogenesis, where there is methylation of the CpG island promoter regions of the tumor suppressor gene* BRAF*, as well as* KRAS* mutation, resulting in the epigenetic silencing of a number of genes [4, 5]. When the* hMLH1* repair gene is inactivated, there is rapid development of cytological dysplasia, potentially followed by malignant transformation. The rapid rate of progression to cancer via the serrated pathway of carcinogenesis may have an important impact on colorectal cancer screening strategies.

The incidence of SSA in the appendix is unknown, but a review of the literature revealed that fewer than 50 cases have been described [3–12]. This report describes a case of traditional serrated adenoma of the appendix with an intraepithelial carcinoma removed by laparoscopic resection of the appendix and part of cecal wall, where the diagnosis was confirmed by a histopathological study.

## 2. Case Report

**Figure 1:**
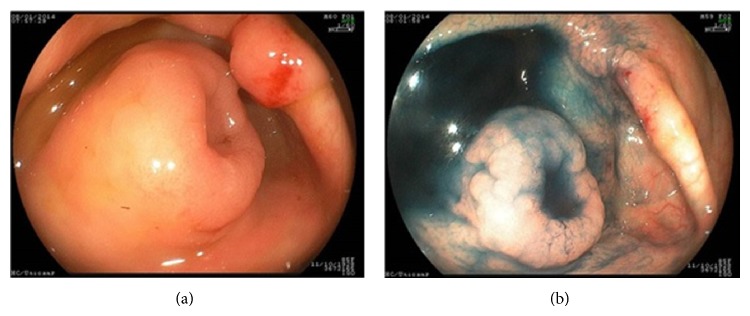
(a) A colonoscopic view of the protruding adenoma surrounding the ostium of the appendix. (b) The same image after chromoscopy with indigo carmine.

An 86-year-old woman was referred for a screening colonoscopy because she complained of loss of weight associated with diarrhea and anemia. The patient was in regular general condition, and a physical examination revealed pain on deep palpation of the lower right abdomen. The family reported that the patient had undergone a subtotal gastrectomy to resect an early adenocarcinoma of the gastric antrum 22 years earlier and reported no prior family history of colorectal cancer. The family indicated that the patient started taking cholinesterase inhibitors for the treatment of initial Alzheimer's disease beginning about two years earlier. The colonoscopy identified left-side diverticular disease and the presence of a vegetating lesion protruding into the cecal lumen from the appendicular ostium. The lesion was reddish with a fine nodular surface that spread to the wall of the cecum, with a length of 1.5 cm around the ostium of the appendix (Figures 1(a) and 1(b)).

Chromoscopy with nebulization of indigo carmine showed that the mucosal surface of the cecum was uneven and that there was no clear demarcation regarding its limits. The glands had star-shaped pits similar to conventional type II pits but, in focal areas, this pattern of crypts changed to type IV of Kudo classification [13]. The ostium of the appendix was located in the central portion of the lesion, which was covered with a thin layer of mucus, and had no apparent signs of inflammation or obstruction. Abdominal computed tomography could not identify the appendicular mass or any lymph node enlargement in the abdominal cavity.

**Figure 2:**
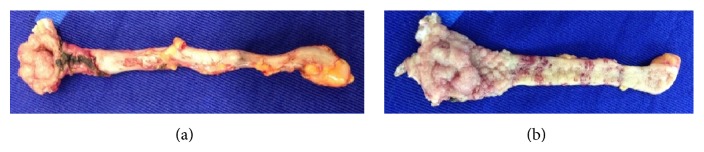
(a) An external view of the surgical specimen of the appendix showing the serrated adenoma protruding through the ostium of the appendix. (b) The internal view of the appendix lumen showing the adenoma arising in the appendix mucosa and spreading to the cecum wall.

Based on a suspicion of an adenoma of the vermiform appendix, fragments were collected for a histopathology study. Microscopic analysis showed the presence of a tubular adenoma with low-grade dysplasia (Vienna 3) [14]. Due to the characteristics and location of the lesion, which did not allow safe endoscopic removal during colonoscopy, the patient was referred for laparoscopic resection. The laparoscopy showed that the tumor began in the proximal third of the appendix, and nearly was implanted in the cecum, without compromising the serosa of the appendix and cecum and without any lymph node enlargement. Considering the advanced age of the patient, a resection of the appendix and part of the cecal wall was performed with a single firing of a laparoscopic mechanical linear stapler (Figure 2). The surgical specimen was removed out of the abdominal cavity inside a plastic bag.

**Figure 3:**
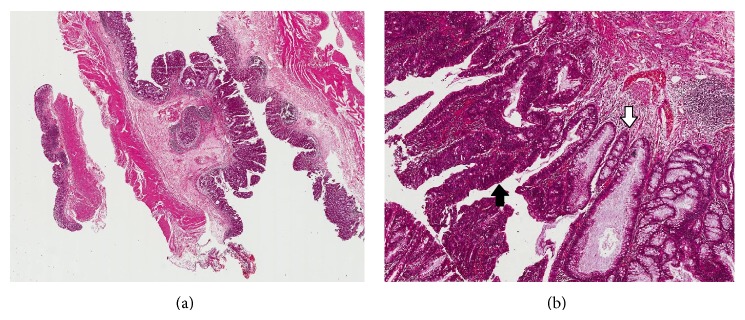
(a) The traditional sessile serrated adenoma of the appendix. (b) The intramucosal adenocarcinoma (black arrow) arising in the traditional sessile serrated adenoma (white arrow).

The entire surgical specimen was subjected to a histopathological study. The macroscopic examination showed the presence of a circumferential vegetating mass located in the vermiform appendix, with 2 cm of length that protruded into the cecal ostium of the appendix. The lesion compromised 1.5 cm of the cecum wall. The histological sections showed the vermiform appendix with a sessile polypoid lesion characterized by the proliferation of the epithelial lining with a lush serrated architecture. The tissue slices showed the presence of dilated crypts compressing the lamina propria at the base of the lesion. The epithelial cells presented eosinophilia of the cytoplasm and light atypia with tubular glands that were intensely stained, elongated, and slightly stratified. In focal areas, there were glandular structures lined by epithelial cells with marked atypia and complex architectural changes. The margins of surgical specimen were free of neoplastic cells. These findings led to a diagnosis of intramucosal carcinoma originating in a sessile serrated adenoma of the appendix (Figure 3). After surgery, the patient showed an uneventful postoperative course and was discharged on the fourth day. One year after the surgical procedure, the patient had recovered from the weight loss and anemia.

## 3. Discussion

Neoplastic lesions of the vermiform appendix are still considered to be rare, with an estimated incidence of 0.08% to 0.1% of all appendiceal specimens [15–18]. Graham et al. reviewed 6,824 surgical specimens of vermiform appendices in 2009 and found 42 primary epithelial tumors, yielding a prevalence rate of approximately 0.62%, with subjects having a mean age of 45.9 ± 19.3 years and with a male-to-female ratio for all tumors of 1 : 1 [19]. Usually, neuroendocrine tumors, adenomas, and adenocarcinomas comprise the majority of these tumors, with reported incidences of 47.6%, 45.2%, and 7.14%, respectively [19]. Much less commonly, lymphomas and sarcomas can also affect the appendix, with respective incidences of 1.7% and <1% [20]. Among the main polyps that develop in the vermiform appendix, adenomas (villous, tubular-villous, and tubular) and serrated polyps (SPs) are the most common and deserve special attention due to the possibility of malignant degeneration to cancer. The preoperative diagnosis of polyps of the appendix is difficult, and a review of such cases showed that more than 93% were diagnosed after an appendectomy or at autopsy [12, 19, 21, 22]. Occasionally, these lesions are found incidentally while performing an ultrasound or computed tomography study to evaluate other conditions of the abdominal cavity [19].

SPs of the vermiform appendix are a heterogeneous group of lesions comprising hyperplastic polyps (HPs), sessile serrated adenomas/polyps (SSA/Ps), and traditional serrated adenomas (TSAs) [18]. The term SPs is used for polyps with a serrated aspect of the epithelial surface and crypt epithelium, which most likely occurs due to an increase in the cellular proliferation in the crypt zone, as well as due to inhibition of programmed cellular death of the specialty cells located on the top of the colonic glands [4]. A review of the literature revealed that only one case was published before 2004 [6] and fewer than 50 cases have been described to date [3–12].

The correct classification of SPs of the appendix remains confusing due to the rarity of the disease [23, 24]. In 1990, Longacre and Fenoglio-Preiser proposed that all lesions with a serrated architecture and atypia should be classified as SA [1]. Torlakovic and Snover [24] subsequently suggested the existence of a subtype comprising atypia admixed within HPs, for which the name SSA was proposed. In 2004, Rubio [3] reviewed a total of 38 noncarcinoid, nonneoplastic, or neoplastic polyps or tumors of the appendix and classified these polyps into three distinct histological types. According to the author of that study, there exists in HPs a mucosal hyperplasia in which the crypts show a sawtooth configuration as a result of crenate epithelium; the cells are columnar with or without apical mucous vacuoles alternating with large goblet cells, and the bases of the crypts are lined by regular cells with small round nuclei [3]. In the villous type, adenomas have >80% of the dysplastic epithelium arranged as straight villous fronds [3]. Finally, in serrated adenomas, the lesion has crenate, sawtooth-like structural changes in the dysplastic epithelium covering the basal aspect of the crypts as a result of epithelial infolding [3]. Recently, the World Health Organization (WHO) proposed a new classification for the family of SPs, dividing them into HPs, SSA/Ps, and traditional serrated adenomas (TSAs) [25]. The histopathological findings of the patient in the present report suggest that the appendiceal polyp had characteristics of a TSA.

HPs are the most common members of the SPs family and can be found throughout the colon and rectum, but with distal predominance. HPs are characterized by their simple elongated crypt architecture and narrow crypt bases resembling normal mucosa, with proliferative activity confined to the deep area of the colonic glands [4]. SSA/Ps less frequently affect the proximal colon. The diagnosis of SSA/Ps is based mainly on histological characteristics, including serration, dilatation, horizontal orientation, an L-shaped or inverted T-form at the base of the crypts with an asymmetrical proliferative zone, and goblet cell differentiation [4]. TSAs, the last and rarer type of SPs, have a protuberant growth pattern with a complex villous configuration and premature crypt formation, defined as “ectopic crypts” [4]. Studies suggest that there are histological differences between SPs located in the vermiform appendix and colon [4, 9]. Bellizzi et al. [9], as well as the present case, showed that SSA/Ps have a greater tendency to affect the entire circumference of the appendix mucosa, while with HPs, the mucosal involvement is more focal [9]. Yuyucu Karabulut et al. [4] have studied SPs of the appendix, and report that basal dilatation, basal serration, T-/L-shaped crypts, and ectopic crypts are significantly more common in SSA/Ps than in HPs. SSA/Ps and TSAs seem to be more susceptible to malignant transformation, and it was verified that dysplasia was observed in 31.4% of SSA/Ps, while HPs did not show dysplasia [4].

The importance of SPs has increased in the last decade because morphological and molecular studies have determined that this type of lesion develops though the serrated pathway of colorectal carcinogenesis or due to the CpG island methylated phenotype pathway (CIMP pathway). It is estimated that at least 15% of all CRC may be related to the serrated pathway [26]. This newly described pathway of cancer is associated with a rate of malignant transformation greater than that of the other two classical pathways of colorectal cancer [9].

The serrated pathway is characterized by an epigenetic mechanism that involves abnormal methylation of CpG islands in the promoter regions of tumor suppressor genes and may be associated with mutations of the* BRAF* oncogene. Mutations in this gene play the equivalent role that* KRAS* mutations play in chromosomal instability colorectal cancer [26]. In the CIMP pathway of colorectal carcinogenesis, a SSA/P (with a* BRAF* mutation and extensive CpG methylation) develops cytological dysplasia and ultimately invades as a high microsatellite unstable cancer (MSI-H) [9]. This new colorectal carcinogenesis pathway differs significantly from the other two classic routes of carcinogenesis described previously (the adenoma-carcinoma sequence and microsatellite instability). However, there are still many aspects that are unclear regarding the serrated pathway for SSAs of the appendix compared to those with colonic localization [23, 24]. Within the serrated pathway, there is also a possibility that there may be methylation of* hMLH1*, which is associated with SSA/Ps with severe dysplasia. These lesions with mismatch repair gene mutations and severe dysplasia are considered to progress more rapidly to CRC. A recent study that analyzed mutations in the* KRAS/RAF/MAPK* pathway in 132 appendiceal lesions verified that SSA of the appendix often harbors* KRAS* mutations, rather than* BRAF* mutations, confirming that the serrated pathway in the appendix is likely different than in the colon and rectum [5]. Likewise, other authors have showed that MSI-high appendiceal carcinomas highlight the low prevalence of MSI in the appendix compared to the right colon and suggest that* hMLH1* promoter methylation is not a major mechanism underlying microsatellite instability in this location [27].

No typical signs or symptoms could be regarded as particularly representative of appendiceal polyps [16]. The most common clinical presentation reported in the medical literature is acute appendicitis. The narrow appendiceal lumen may be occluded by the tumor early during the course and predisposes it to inflammation and perforation [7, 11, 28]. Other possible manifestations included a palpable abdominal mass, ascites, carcinomatosis and peritonitis resulting from a perforated appendix, and a variety of nonspecific symptoms [16]. Rarely, SSA of the appendix was shown to lead to an intussusception of the appendix [10, 12]. Usually, the above presentations were incidental findings during intra-abdominal surgery, during abdominal CT scans for other medical conditions, or during a colonoscopy performed to screen for colorectal cancer, as in the patient in this report. The difficulty of a colonoscopy-based diagnosis lies in the fact that these lesions are small and located within the appendix. The lumen of the appendix has a reduced dimension that prevents the access of the colonoscope. If the neoplastic tissue affects the ostium of the appendix and reaches the mucosa of the cecum, as what occurred in the present patient, the diagnosis is easier to establish. The mucosa of the cecum is generally convex and protrusive, and the ostium of the appendix is located in the center of the lesion, resembling the crater of a volcano (volcano sign). In most cases, as in our patient, the ostium may be covered by a superficial mucus layer [29]. When the tissue is nebulized with indigo carmine, chromoendoscopy and magnification images show pit patterns with star-shaped pits similar to conventional type II pits, but with dilated openings of the glandular crypts, uniform pits with serrated architecture classified as a type II-O pit pattern. However, there were small areas near the ostium of the appendix where the pattern of pits was modified to the IVs type usually found in TSAs [29].

Because of the potential risk of malignant transformation, the most prudent option for treatment of adenomas located in the vermiform appendix is the complete resection of the polyps with free margins. Whether the resection should be managed by right hemicolectomy or appendectomy is controversial [30]. Some authors suggest simple appendectomy for carcinoma confined to the mucosa or well-differentiated adenocarcinoma with submucosal invasion [31]. In cases where there is a compromise of the cecal wall by the adenoma, as was found in the present report patient, resection of the appendix and part of the cecum wall may be sufficient. This option may also be indicated in patients of advanced age because such patients have shown a worse clinical performance. Right colectomy with regional lymphadenectomy is indicated for patients with a large adenomas suspected to have malignant degeneration or for a tumor involving the cecum or adjacent organ [32]. Although colonoscopy can remove polyps of the appendix by piecemeal resection, neoplastic lesions, some lesions, particularly those that are invasive, should still be completely resected with sufficient margins [33]. In some patients, the precise endoscopic definition of the site of origin (cecal or appendiceal) may be difficult if the appendiceal base appears to be the principal area of neoplastic change because complete surgical excision is essential [32]. The appendix resection may be performed by the surgeon through conventional laparoscopic techniques. Laparoscopy is associated with less surgical trauma and faster postoperative recovery than other approaches.

In the patient described in this report, we opted to perform laparoscopic resection in order to minimize the surgical trauma in our patient with neurological disease. During the laparoscopic procedure, we verified that the lesion was confined to the proximal third of the appendix and a small distal part of the cecum wall. The serosa of the appendix and cecum showed no signs of infiltration by the neoplasia, and no regional enlarged lymph nodes were detected. These findings encouraged us to carry out the removal of the appendix and the cecal wall by this type of access. After the longitudinal opening of the surgical specimen, the colonoscopic findings could be confirmed, and we could observe that the surgical resection margins were approximately 2 cm from the lesion. The histopathological study, which showed an intramucosal carcinoma (*in situ* carcinoma), confirmed that the surgical resection was adequate.

In summary, we experienced a rare case of a traditional serrated adenoma with intramucosal carcinoma involving the appendix that could be diagnosed by colonoscopy before surgery. This case reinforces the importance of carefully inspecting the cecum and appendiceal orifice during a screening colonoscopy in order to detect appendiceal tumors.
